# The Treatment of Intermittent Convergent Strabismus in Children by Mydriatics or Myotics

**Published:** 1880-01

**Authors:** Lucien Howe


### Translations.

### THE TREATMENT OF INTERMITTENT CONVERGENT STRABISMUS IN CHILDREN BY MYDRIATICS OR MYOTICS—BY DR. BOUCHERON.

FROM THE FRENCH, BY LUCIEN HOWE, M. D.

In the great majority of cases, as Donders has shown, strabismus depends upon the hypermetropic form of the eye.

Persons with such eyes must exert their accommodation even for distant objects, and are obliged to make excessive efforts to see those near at hand.

But the eye is so adjusted that any accommodation for a near object necessitates a convergence of the axes of vision toward that point. The muscles of accommodation and of convergence are excited by the same nerve (motor oculi communis), and these two actions are therefore "associated." The accommodation, however, *controls* convergence, which is really a secondary action, and if an unusual effort is made in accommodating, excessive convergence inevitably follows.

Now, hypermetropic eyes, being so constructed that they are obliged to accommodate strongly, are continually called upon to converge unnaturally, in other words to be "crossed." And this is the case at first, momentarily, when actuated by unusual efforts of accommodation or by some cerebral excitement; and afterwards the habit is established, the intermittent strabismus then becomes permanent, the muscles having contracted in an abnormal position, and being thus rendered fixed, the condition is curable only by an operation.

But, while the strabismus is still intermitting, when the habit is not yet fully developed, it is possible to obviate the strabismus by meeting the indications.

The excessive accommodation and the unnatural shape of the eye are, as we have seen, the two prime factors originating the strabismus.

We must, therefore, act on these. Inasmuch as accommodation regulates the convergence, and excessive accommodation necessitates abnormal convergence, let us do away with accommodation, and at the same time we suppress the unnatural convergence and the strabismus.

Nothing is more simple. A solution of atropine dropped into the eyes, paralyzing the accommodation, also arrests the tendency to convergence, and in a few days (from ten to fifteen) the intermittent strabismus will have disappeared.

As an equilibrium between the ocular muscles is thus established, the natural development and growth of the child will render it more fixed, and after some months—three, five, eight or ten, according to the age of the child—the cure will be permanent. As in the majority of cases, the convergent strabismus is at first intermittent, it appears that this method is applicable to a larger number.

After the disappearance of the strabismus, it is important to correct the unnatural shape of the eye, in other words, the hypermetropia, by means of appropriate glasses. This is especially necessary when the child is of such an age as to use its eyes continually in study.

All the mydriatics, atropine, duboisine, etc., have about the same advantages, when thus used. Myotics, such as esserine, can also be used, for they contract the ciliary muscle firmly, and thus disturb the pre-existing relation between accommodation and convergence; but they are preferable toward the end of the treatment when attempts are made at reading. These agents, when employed in suitable doses, are absolutely free from danger, even to young children.—*Union Medicale* (*Annales d'oculistique*).

